# Toll-Like Receptors, Tissue Injury, and Tumourigenesis

**DOI:** 10.1155/2010/581837

**Published:** 2010-09-14

**Authors:** Savvas Ioannou, Michael Voulgarelis

**Affiliations:** Department of Pathophysiology, Medical School, National University of Athens, 75 Mikras Asias Street, 11527 Athens, Greece

## Abstract

Toll-like receptors (TLRs) belong to a class of molecules known as pattern recognition receptors, and they are part of the innate immune system, although they modulate mechanisms that impact the development of adaptive immune responses. Several studies have shown that TLRs, and their intracellular signalling components, constitute an important cellular pathway mediating the inflammatory process. Moreover, their critical role in the regulation of tissue injury and wound healing process as well as in the regulation of apoptosis is well established. However, interest in the role of these receptors in cancer development and progression has been increasing over the last years. TLRs are likely candidates to mediate effects of the innate immune system within the tumour microenvironment. A rapidly expanding area of research regarding the expression and function of TLRs in cancer cells and its association with chemoresistance and tumourigenesis, and TLR-based therapy as potential immunotherapy in cancer treatment is taking place over the last years.

## 1. Introduction

Toll-like receptors (TLRs) are a family of transmembrane receptors that play a key role in the nonspecific or innate immune defense, particularly in inflammatory response against various invading exogenous pathogens, by recognising receptor-specific pathogen-associated molecular patterns of highly conserved pathogenic components of bacteria, viruses, fungi, and parasites [1]. Once these pathogens have breached physical barriers such as the skin or intestinal tract mucosa, they are recognized by TLRs which activate immune cell responses against their structurally conserved molecules. The most important role of TLRs in host defense is the regulation of innate and adaptive immune responses by epithelial cells, the first line of defense at mucosal sites such as the respiratory, genitourinary, and gastrointestinal tracts, and the skin. TLRs also have a crucial role in mediating leukocyte recruitment to infected tissues and the uptake of microorganisms by phagocytic cells [2, 3]. Activation of antigen-presenting cells (APCs) such as dendritic cells (DCs) and stimulation of both T- and B-cell-mediated immune responses are due mostly to ligation of TLRs [4].

Moreover, TLRs have an important role in maintaining tissue homeostasis by regulating tissue repair and regeneration. TLR ligands in this case can be either microbial (exogenous) or host derived (endogenous) [5]. In addition, TLR signalling has also been shown to regulate apoptosis with the expression of antiapoptotic proteins or inhibitors of apoptosis.

They derive their name from their similarity to the protein coded by the Toll gene identified in Drosophila in 1985 by Christiane Nüsslein-Volhard [6]. The human TLR family consists of currently ten members, which are structurally characterized by the presence of a leucine-rich repeat (LRR) domain in their extracellular domain and a Toll/Interleukin (IL)-1 receptor (TIR) domain in their intracellular domain [7]. Epidemiologic studies of the many single nucleotide polymorphisms that have been identified in various TLR genes have been studied in relationship to tumourigenesis. Studies of common genetic variants in various populations and growth stimulation of cancer cell lines suggest that TLR4 plays an important role in the development of H. pylori-associated gastric cancer [8, 9]. Other studies have found that common polymorphisms in TLR2 are associated with increased risk of colorectal cancer [10], gastric cancer [11] and lymphoma [12], while a specific haplotype in TLR10 is associated with increased risk of nasopharyngeal cancer [13]. A recent study revealed a significant association of a common genetic variant within the TLR10-TLR1-TLR6 gene cluster with decreased risk of initial development of prostate cancer [14].

The relationship between inflammation and tumourigenesis and tumour progression is widely accepted. Numerous studies have provided convincing evidence that bacterial- and viral-induced inflammatory process can mediate tumourigenesis. Moreover, it is well known that regular intake of nonsteroidal antiinflammatory drugs lowers the risk of developing some types of cancer. 

Due to the important role in inflammation and tissue regeneration, TLRs are likely candidates to mediate effects of the innate immune system on tumourigenesis. TLR expression and function in cancer cells and its association with the inflammation process and tumourigenesis will be discussed here.

## 2. Toll-Like Receptors Signalling Pathways

TLRs belong to a class of molecules known as pattern recognition receptors. The ligands for these receptors are components of pathogenic microbes and are often called pathogen-associated molecular patterns (PAMPs). The recognition of PAMPs by TLRs is a cornerstone of innate immunity and provides a quick and highly efficient response to pathogens in both vertebrate and invertebrate species [15]. In addition to recognizing ligands derived from foreign microbes TLRs, in particular TLR2 and TLR4 have been reported to bind numerous endogenous ligands termed damage-associated molecular patterns (DAMPs) that are released from injured and inflamed tissue [5]. Thus, the TLR-mediated immune response may be activated in the absence of foreign microbes. Upon binding of a microbial ligand, TLRs activate signalling pathways that stimulate cytokine production and other parts of the innate immune response. Well-known TLR microbial ligands are lipopolysaccharide (LPS), a membrane component of Gram-negative bacteria, bacterial lipoproteins, lipoteichoic acid and fungal zymosan, bacterial flagellin, double-stranded RNA, and single stranded RNA. TLR4 senses Gram-negative bacteria by binding LPS. Bacterial lipopeptides and lipoteichoic acids (LTAs) stimulate TLR2 responses. TLR3 and TLR7 sense viral infections by recognizing double-stranded and single-stranded RNA, respectively. TLR9 recognizes nonmethylated CpG-containing DNA from bacteria and viruses. 

TLRs that mainly serve to detect bacterial LPS and lipoproteins are located on the cell surface (TLR1, TLR2, and TLR4-6) whereas those that mainly recognize viral RNA and bacterial DNA are located in late endosome-lysosomes in which these materials are processed (TLR3, TLR7, TLR8, and TLR9). TLR1, TLR2, TLR4, and TLR6 initiate signalling by heterodimerization. TLR2 forms heterodimers with TLR1 or TLR6 which recognize bacterial triacylated and diacylated lipoproteins, respectively [7]. A wide range of DAMPs including heat shock proteins, high-mobility group box 1 (HMGB1), uric acid crystals, hyaluronan, heparin sulfate, messenger RNA, surfactant protein A, and various products of the extracellular matrix such as fibronectin and fibrinogen have been suggested to activate TLRs [16, 17]. 

**Figure 1:**
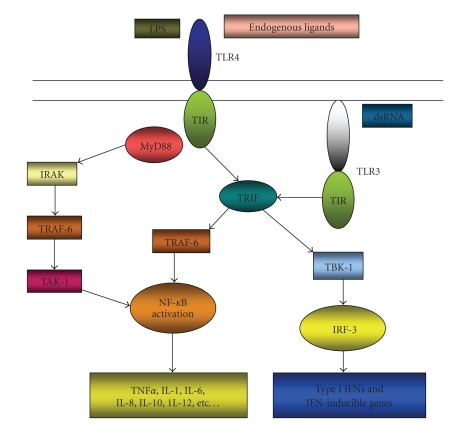
Toll-like receptor (TLR) signalling pathways. Membranal TLRs (represented by TLR4) recognize external ligands (exogenous and endogenous), while cytoplasmic TLRs (TLR3) recognize intracellular signals. When activated, the majority of TLRs induce activation of NF-*κ*B (early phase of NF-*κ*B activation) and cytokine production in a MyD88-dependent manner; while TLR4, like TLR3, can also signal in a MyD88-independent manner and induce the expression of type I interferons (IFN) and IFN-inducible proteins in addition to a late phase NF-*κ*B activation.

After ligation of TLR ligands, TLRs dimerize and transmit signals throughout the cell through one or more of four adaptor proteins: myeloid differentiation primary response gene 88 (*MyD88*), toll/interleukin-1-receptor-domain-containing adaptor inducing interferon-*β* (*TRIF*), toll/interleukin-1-receptor-domain-containing adaptor protein (*TIRAP*), and TRIF-related adaptor molecule (*TRAM*). Whereas MyD88 is part of the signalling cascade of all TLRs except for TLR3, TRIF only interacts with TLR3 and TLR4 (Figure 1). 

**Figure 2:**
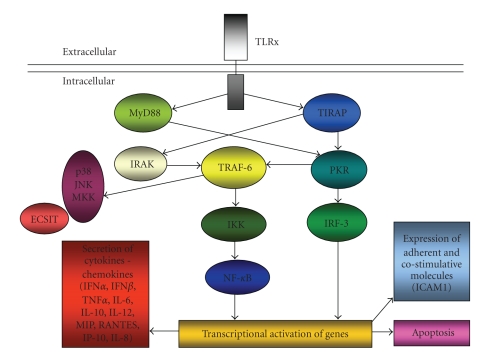
Toll-like receptor (TLR) signalling is mediated by at least two distinct pathways. After recognition of a pathogen-specific molecular pattern, TLRs are capable of differentially activating distinct downstream signalling events via different cofactors and adaptor proteins mediating diverse immune responses [1]. The MyD88-dependent TLR signalling pathway is activated via the conserved, cytoplasmic TIR domain, which provides a scaffold for recruitment of the adaptor molecule MyD88 and serine/threonine kinases of the IL-1R-associated kinase (IRAK) family. Following IRAK autophosphorylation, the TRAF6 adaptor protein interacts and induces translocation of the transcription factor NF-*κ*B to the nucleus, resulting in transcriptional activation of genes encoding cytokines and chemokines (TNF-*α*, NO, COX-2, SOCS (for “suppressor of cytokine signalling”), IP-10, IFN-b and IL-1, 6, 8, 10, 12). In addition, expression of proteins involved in apoptosis and production of adherent and costimulative molecules such as ICAM1, occur. Moreover, TLRs bridge the signalling pathway via ECSIT (for “evolutionarily conserved signalling intermediate in Toll pathways”) to TRAF6 for p42/p44 mitogen-activated protein kinase (MAPK) kinase (MKK), p38, and JNK in response to specific bacterial products [2]. The MyD88-independent TLR signalling pathway is activated via TIRAP and results in activation of the dsRNA-binding protein kinase PKR. This protein has been proposed to be a central downstream component of both the TIRAP- and MyD88-dependent signalling pathways and could mediate potential crosstalk between them. The MyD88-independent pathway appears to utilise both IFN-regulatory factor 3 (IRF3) and NF-*κ*B, and results in the expression of IFN-g-inducible genes including IP-10.

TLR2 or TLR4 agonists stimulate the MyD88 signaling pathway in APCs such as macrophages and DC, which leads to subsequent downstream activation of the major transcription factors, the nuclear factor of kappa light polypeptide gene enhancer in B-cells (NF-*κ*B) and mitogen-associated protein (MAP) kinase signaling pathways (such as the ERK-CREB pathway, the JNK-AP1 pathway, and the p38 pathways) [18]. This leads to the rapid expression of inducible nitric oxide synthase (iNOS) and a wide variety of proinflammatory cytokines, chemokines, and their receptors, including tumor necrosis factor alpha (TNF-*α*), interleukin (IL)-1*α*, IL-1*β*, IL-1ra, IL-6, IL-8, IL-10, IL-12p40, IL-23, and macrophage inflammatory protein (MIP)-1*α*, and MIP-1*β* (Figure 2) [19, 20]. These factors initiate the inflammatory response, increase vascular permeability, direct DC and macrophage migration from the periphery to the central lymphoid organs, and regulate various aspects of adaptive immunity development. 

Recently, naturally arising regulatory T-cells (Tregs) have been shown to express TLRs. Tregs originate from the thymus and are characterized by the expression of Foxp3 as a key control gene for their development and function. Their pivotal role is maintaining immunological self-tolerance. Recent data suggest that the activation of TLRs on Tregs can increase or decrease their suppressive activity, thus providing an important link between innate and adaptive immune responses [21]. Milkova et al. suggested an important role of the NF-*κ*B signalling pathway for the induction and modulation of suppressive function of Tregs, if they are confronted with TLR4 ligands such as LPS [22].

## 3. Toll-Like Receptors, Tissue Repair, and Fibrogenesis

The tissue repair and regeneration process has been reported to depend on MyD88 signalling. The importance of this signalling pathway has been demonstrated by a recent study which showed that wound healing was impaired in MyD88-deficient mice [23]. MyD88 is a critical signal adaptor for TLRs 2, 4, 5, 7, 8, 9, and 11 whereas TLR3 signals in a MyD88-independent manner through the TRIF adapter pathway.

TLR activation in wound healing appears to be mediated by two entirely different classes of ligands: (1) in organs that are in direct contact with microbiota (intestine, skin, liver), tissue injury leads to a breakdown of protective barriers and subsequent TLR activation by bacterial PAMPs. (2) In many organs such as liver, heart, and kidney, tissue injury leads to release of DAMPs from dead and dying cells resulting in the activation of TLRs and “sterile inflammation.” The release of endogenous TLR ligands predominantly occurs after massive tissue injury, especially where a significant percentage of cells undergo necrosis such as in ischaemia-reperfusion injury. Activation of TLRs modifies tissue injury in positive or negative fashion either by recruiting inflammatory cells that release cytotoxic mediators or by activating cytoprotective signals. TLRs exert a cytoprotective role and prevent tissue injury in the lung and the intestine. In bleomycin-induced lung injury, TLR2-TLR4- and MyD88-deficient mice display an increased degree of lung injury despite reduced recruitment of inflammatory cells. Jiang et al. showed that by blocking the endogenous ligand hyaluronan by a peptide-based approach, the pattern of lung injury was highly similar to that of MyD88 and TLR2-TLR4-deficient mice [24].

In contrast, ischaemia-reperfusion injury represents the scenario in which a profound injury-promoting role of TLR2 and TLR4 has been most thoroughly established. Several studies have shown the protection of TLR4-mutant or deficient mice after hepatic and cardiac ischaemia-reperfusion [25, 26]. DCs are the most likely candidate to mediate injury following ischemia-reperfusion injury. After hepatic ischemia-reperfusion, wild-type but not TLR4-mutant dendritic cells displayed a strong increase of TNF-*α* production [27], a well-known mediator of hepatic ischemia-reperfusion injury that is rapidly and potently released from macrophages and DC following TLR activation [28, 29]. Therefore, the most likely scenario is that HMGB1 is released from necrotic parenchymal cells to activate TLR4 on DC which in turn releases TNF-*α* to promote tissue injury.

TLR receptors are also involved in the regulation of epithelial proliferation and angiogenesis following injury. TLR4- and MyD88-dependent signals following dextran sulfate sodium-(DSS-) mediated colonic injury, are required to induce cyclooxygenase 2-(Cox2-) mediated generation of prostaglandin E2 (PGE2) and to stimulate epithelial cell proliferation [30]. The MyD88-Cox2 signal that promotes regeneration is largely provided by macrophages which migrate toward the site of injury to stimulate the proliferation of epithelial progenitors. PGE2 is one crucial growth-promoting signal provided by stimulated macrophages. The mechanism for improved epithelial repair may be through PGE2-dependent activation of epidermal growth factor receptor (EGFR) [31]. The TLR4-MyD88 signaling axis is also involved in the regulation of angiogenesis to restoring blood flow to the site of injured tissue. Promotion of angiogenesis by TLR4 appears to be restricted to specific pathophysiological circumstances. In skin wounds, absence of MyD88 results in slower wound healing, decreased angiogenesis, and decreased vascular endothelial growth factor (VEGF) [23]. TLR4 activation alone is not sufficient for the induction of VEGF, but requires the presence of adenosine, and is largely mediated by a transcriptional upregulation of HIF1*α* which binds to a known hypoxia response element in the VEGF promoter [32].

Moreover, there is accumulating evidence that TLRs directly target fibroblasts to induce activating signals. One main mechanism by which TLRs modulate fibrogenic responses is through the transforming growth factor beta (*TGF* *β*) signaling pathway. In the liver, TLR4 and MyD88 are required for the development of fibrosis in chronic hepatitis. Activation of TLR4 sensitizes hepatic stellate cells, the main precursor of fibroblasts in the liver, toward the effects of TGF*β* and thereby, promotes collagen production [33]. This effect is mediated by the downregulation of an inhibitory TGF*β* pseudoreceptor, Bambi. Bambi downregulation is mediated through a MyD88-dependent and TRIF-independent pathway [33]. In addition, TLRs promote proinflammatory and antiapoptotic signals in fibroblast populations through NF-*κ*B [34, 35].

## 4. Toll-Like Receptors and Apoptosis

Toll-like receptors are potent activators of the NF-*κ*B pathway [4]. NF-*κ*B regulates the transcription of a number of antiapoptotic genes such as Bcl-2, iNOS, c-FLIP, inhibitor of apoptosis (IAP), and TRAF molecules [36]. There is increasing evidence that TLRs provide signals to promote the survival of epithelial cells under stress conditions. It has already been demonstrated that TLRs exert a cytoprotective role in the lung and the intestine [24]. Additionally, TLR4-MyD88-NF-*κ*B-Cox2 axis is involved in protection from apoptosis in normal as well as premalignant cells of the colon [30]. Cox2 is a known mediator of antiapoptotic, proliferative, and tumor-promoting effects in the colon as mentioned above [30].

Moreover, TLRs assist natural killers (NK) cells in the killing of infected cells either by direct stimulation on NK cells or the induction of type I interferons and IL-15. Becker et al. found that Leishmania lipophosphoglycan (LPG) activates NK cells through TLR2 [37].

## 5. Toll-Like Receptors and Tumourigenesis

Several studies indicate that TLR signalling contributes to the growth of tumours in numerous organs. The development of cancer has been associated with microbial infection, injury, inflammation, and tissue repair. The subsequent activation of TLRs in cancer cells and the ensuing signalling cascade with the cytokine/chemokine production may promote cancer cell survival, chemoresistance and therefore tumour progression. In a recent study, Huang et al. reported the expression of TLR4 in murine tumour cell lines and showed that the activation of TLR4 in these tumour cells by LPS induced tumour evasion from immune surveillance [38]. In another report it has been shown that in a subgroup of epithelial ovarian cancer cells that express MyD88, ligation of TLR4 by LPS induced cell proliferation and enhanced cytokine/chemokine production [39]. Huang et al. also showed that Listeria monocytogenes promotes cell growth through TLR2 [40]. In 2007, He et al. described the expression of TLR4 in human lung cancer cells [41]. The direct promotion of cancer cell survival and the inflammation-induced chemoresistance have been linked to the hyperactivation of NF-*κ*B in cancer cells, which induces upregulation of antiapoptotic proteins such as c-FLIP and XIAP, and inhibition of proapoptotic proteins such as Bax and Caspase-9 [42, 43]. Another report showed that the ligation of TLR2 in lung cancer cells induced the activation of mitogen-activated protein kinases (MAPK) as well as NF-*κ*B which were shown to prolong cancer cell survival [40].

A number of recent studies have investigated carcinogenesis in mice deficient in TLRs or TLR adapter molecules using models of inflammation-associated cancer. These models provide a physiological tumour environment thus accounting for a possible role of TLRs in stroma-tumour interactions, possibly mediated by endogenous TLR ligands released from necrotic tumour cells. TLR4-deficient mice display a profoundly reduced dysplasia, number and size of tumours [44]. Similar results were reported in a study that investigated the role of MyD88 in colon cancer. MyD88 deficiency leads to decreased carcinogenesis in noninflammatory model of colon cancer [45]. Additionally, the development of fibrosis-associated hepatocellular cancer in patients with chronic hepatitis, it is likely that is promoted by the TLR-MyD88 pathway [33].

 Another interesting hypothesis is that cancer development might be an abnormal form of tissue repair in which the control mechanism loses its function. The presence of deregulated infection, inflammation and/or tissue injury as occurs during various stages of tumourigenesis, leads to unregulated TLR-regulated tissue repair response [46]. MicroRNAs (miRNAs) are a class of small RNA molecules that regulate gene expression at posttranscriptional level and may function as either oncogenes or tumour suppressors. Several studies have demonstrated a link between miRNAs and TLR function, and therefore potential association with inflammation and cancer formation. *MiR-155*, one of the most studied miRNAs related to inflammation and cancer, is highly expressed in B-cell lymphoma, breast and lung cancers, and pancreatic adenocarcinomas. A recent study showed that ligands for TLR2, TLR3, TLR4 and TLR9 could all induce the upregulation of miR-155 expression, through both MyD88- and TRIF-dependent pathways [47]. *MiR-146* is highly expressed in breast, prostate, pancreatic, stomach and papillary thyroid carcinomas, and is a target of NF-*κ*B, upregulated upon TLR2, TLR4 or TLR5 ligation [48].

## 6. Toll-Like Receptors and Tumour Microenvironment

The regulation of immune response within the tumour microenvironment might be another consequence of TLR activation. The infiltrating immune cells contribute to cancer growth and metastasis. In a study on oral epithelial squamous cell carcinoma it has been shown that the level of immune cell infiltration was directly correlated with the level of morphological and pathological transformation from normal to malignant phenotypes [49]. During both cancer development and tissue repair process, the immune infiltrate is characterized by the presence of a high number of macrophages, which produce VEGF, IL-6, IL-10, prostaglandins, iNOS, and IDO. Inflammatory cells, primarily macrophages, are consistently regarded as critical mediators involved in cancer initiation and promotion [50]. They facilitate angiogenesis and extracellular matrix breakdown and remodeling and promote cancer cell invasion.

The tumor stroma is made up of diverse cellular populations including macrophages, lymphocytes, vascular cells, and carcinoma-associated fibroblasts. Versican, a large extracellular matrix proteoglycan, accumulates both in tumour stroma and cancer cells. It participates in cell adhesion, migration, and angiogenesis, all features of invasion and metastasis. A recent study has documented that versican can activate tumour-infiltrating myeloid cells through TLR2 and its coreceptors TLR6 and CD14 and elicit the production of proinflammatory cytokines including TNF-*α* that enhance tumour metastasis [51]. The interaction between versican and TLR2 links inflammation and metastasis. 

As both resident fibroblasts and endothelial cells (ECs) also express functional TLR2 and its coreceptors, versican may trigger the activation of both fibroblasts and ECs, leading to a marked increase in interleukin-8 (IL-8) production [52]. IL-8 is a proinflammatory chemokine with leukocyte chemotactic, tumourigenic, and proangiogenic properties. It increases proliferation and survival of endothelial and cancer cells and enhances the migration of cancer cells, ECs, and infiltrating neutrophils at tumor sites [53]. Interestingly, ligation of TLR2 by versican appears to be directly involved in the activation of multiple types of cells in tumour stroma, including macrophages, and the induction of inflammatory cytokine secretion to generate an inflammatory microenvironment hospitable for tumor progression [54].

## 7. Toll-Like Receptors and Cancer Treatment

It was the original observation that certain cancer patients who developed concurrent bacterial infections would also experience concomitant remission of their malignant disease [55]. It has been suggested that host molecules including TNF, have implicated in the mediation of the antitumour effects of endotoxin [56]. However, subsequent testing using recombinant TNF showed many of the toxicities of endotoxin without however equaling the significant antitumour efficacy in both clinical trials and laboratory studies. Although TNF failed to materialize the antitumour effect, the identification and production of several cytokines supplied the dream of cancer immunologists. However, almost all of several cytokines have been tested in animal tumor models, but none has demonstrated similar antitumour effects of endotoxin, except one: IL-12. Taking into consideration the close similarity of the antitumour characteristics between endotoxin and IL-12, and the fact that endotoxin is able to induce IL-12 production, it has been hypothesized that endotoxin exerts its antitumour effect through the induction and the biological activity of IL-12. Interestingly, exposure of DC to PAMPs like TLR ligands induces DC to produce high levels of IL-12p70 and promote efficient T-cell help [55]. Murine studies also showed that only maturation with TLR ligands generates mature DC enable to produce IL-12 and promote optimal T-cell help [57]. In this regard, it has recently demonstrated that a maturation cocktail combining the TLR3 ligand polyinosinic-polycytidylic acid (poly(I:C)) and the TLR7/8 ligand R848 supplemented with PGE_2_ yields DC with both high migratory capacity and IL-12p70 production upon T-cell encounter [58]. Whether the vaccine-matured DC improves antitumour responses in vivo is yet unknown but all these findings open new roads concerning the role of TLRs in the treatment of neoplastic diseases. 

A recent study suggests that TLR4 and MyD88 play an important role in antitumour responses following chemotherapy and irradiation. TLR4-deficient mice have significantly larger tumours after doxorubicin and oxaliplatin treatment or irradiation than wild-type mice [59]. Data from this study suggests that cell death induction by chemotherapy or irradiation induces the release of HMGB1 to subsequently trigger TLR4 activation in DC, enhance antigen presentation, and promote cytotoxic T-cell responses. The ability of TLR signalling to activate the adaptive immune system has led to attempts to harness this response against cancer cells through the use exogenous administration of TLR ligands. It has been shown that high doses of TLR agonists can lead to apoptosis and directly kill both tumour cells and ancillary cells of the tumour microenvironment, whereas low doses of TLR agonists promote cancer growth [60]. TLR activation may also cause tumours to regress by increasing vascular permeability and by recruiting leukocytes, resulting in tumour lysis by NK and cytotoxic T cells. In addition TLRs are important in the recognition of microbial pathogens such as Epstein-Barr virus, hepatitis B and C viruses, human papilloma virus, and Helicobacter pylori, all of which are important aetiological agents of human cancers. 

TLR2/4 agonists are promising molecules against chemotherapy- or radiotherapy-relapsing tumours. TLR2/4 signalling produces TNF-*α*, which is required for inducing DC maturation and migration [61]. Mature DC that reach the lymph nodes induce a rapid and sustained congestion of lymphocyte traffic and their number determines the magnitude of T-cell proliferation and effector response. In this respect, helper T-cell 2 (Th2) immunity and antibody responses may not be desirable in cancer where Th1 and cytotoxic T-cell responses are necessary [62]. TLR-dependent cytokines play a pivotal role in the establishment and maintenance of the Th1/Th2 balance, with IL-12 and IFN-*γ* committing cells to Th1 lineage differentiation [52, 59]. An IFN-*γ* and IL-12 rich local environment attracts T-cells to the tumor where the rich cytokine milieu promotes the development of a CD4^+^ Th1 antitumor response that eventually gives rise to cytotoxic CD8^+^ antitumor cell response. Conversely, in the absence of IL-12 and IFN-*γ*, IL-4 promotes Th2 development.

TLR2 and TLR4 agonists may differ in their ability to influence Th-cell proliferation [19, 63]. TLR2 preferentially induces IL-10, a cytokine that inhibits the synthesis of several proinflammatory cytokines, and that belongs to the Th2 response in the mouse.

 Conversely, the factors specifically induced by TLR4 (IP-10, IL-12, IL-15, and IFN-*γ*) are all associated with a Th1 lineage commitment. But the picture is somewhat complicated by the observation of Komai-Koma et al. that TLR2 is expressed on activated T-cells as a costimulator for antigen-specific T-cell development and participates in the maintenance of T-cell memory [64].

Moreover, TLR2/4 signaling promotes iNOS-dependent apoptosis of chemotherapy-resistant tumor cell clones. The TLR2/4 agonist, OM-174 is a promising molecule against cancer metastases. Studies in cancer patients showed that intravenous OM-174 induces well-tolerated TNF-*α* secretion, at plasma levels known to permeabilize neoangiogenic tumour vessels to the passage of cytotoxic drugs [65]. 

Mycobacterium bovis bacillus Calmette-Guerin (BCG) is another TLR2/4 agonist effective against superficial bladder tumours [65]. OK-432, a penicillin-killed and lyophilized preparation of a low-virulence strain of Streptococcus pyogenes (group A), is a TLR4 agonist which has been successfully used as an immunotherapeutic agent in many types of malignancies, including head and neck cancer and oral squamous cell carcinoma [66]. OK-432 polarizes the T-cell response to a Th1 dominant state. Tano et al. showed that OK-PSA, an active component of OK-432, induces apoptosis by the activation of caspases through p53-independent pathway via TLR4 signaling in head and neck cancer cells [67]. Imiquimod, a synthetic agonist of TLR7 has been proven very effective as monotherapy for basal cell carcinoma, when applied topically as a 5 percent cream [68]. It is also used in the treatment of actinic keratoses and genital warts.

## 8. Conclusion

TLRs have emerged over the last decade as key regulators of innate and adaptive immune responses. In addition, the TLR pathways have been shown to play a critical role in the regulation of tissue injury and wound healing process as well as in the regulation of apoptosis. However, interest in the role of these receptors in cancer has been increasing. There is a large and growing body of literature exploring associations of TLR biology with tumourigenesis. Common polymorphisms in TLR genes have been associated with increased risk for several types of cancer. TLR activation and regulation of immune response within the tumour microenvironment and the ensuing signaling cascade with the cytokine/chemokine production may promote cancer cell survival, chemoresistance, and therefore tumour progression. Moreover, the unregulated TLR-regulated tissue repair response during various stages of tumourigenesis, and the upregulation of several miRNAs through TLR ligation, might explain the critical role of TLR pathways in cancer progression. Alternatively, increased TLR activation by either microbial or endogenous ligands may stimulate anticancer immunity.

Taken all the above into consideration, targeting the TLRs with TLR agonists might be promising as effective regimens to fight cancer and to prolong survival in cancer patients who relapse under chemotherapy. Thus, better understanding of the function and regulation of the TLR signalling pathways and further study of the effect of TLR activation on cancer cells are essential for the understanding of tumour initiation and progression.
